# A New Culture Model for Enhancing Estrogen Responsiveness in HR+ Breast Cancer Cells through Medium Replacement: Presumed Involvement of Autocrine Factors in Estrogen Resistance

**DOI:** 10.3390/ijms24119474

**Published:** 2023-05-30

**Authors:** Seok-Hoon Jang, Se Hyun Paek, Jong-Kyu Kim, Je Kyung Seong, Woosung Lim

**Affiliations:** 1 Department of Surgery, Ewha Womans University Mokdong Hospital, 1071, Anyangcheon-ro, Yangcheon-gu, Seoul 07985, Republic of Korea; jangsh78@snu.ac.kr; 2 Laboratory of Developmental Biology and Genomics, College of Veterinary Medicine, Seoul National University, 1, Gwanak-ro, Gwanak-gu, Seoul 08826, Republic of Korea; snumouse@snu.ac.kr; 3 Department of Surgery, Ewha Womans University Seoul Hospital, 260, Gonghang-daero, Gangseo-gu, Seoul 07804, Republic of Korea; md.psh1004@ewha.ac.kr (S.H.P.); md069069@ewha.ac.kr (J.-K.K.)

**Keywords:** estrogen, responsiveness, medium replacement, endocrine therapy, aromatase inhibitor, resistance, autocrine factor, breast cancer

## Abstract

Hormone receptor-positive breast cancer (HR+ BC) cells depend on estrogen and its receptor, ER. Due to this dependence, endocrine therapy (ET) such as aromatase inhibitor (AI) treatment is now possible. However, ET resistance (ET-R) occurs frequently and is a priority in HR+ BC research. The effects of estrogen have typically been determined under a special culture condition, i.e., phenol red-free media supplemented with dextran-coated charcoal-stripped fetal bovine serum (CS-FBS). However, CS-FBS has some limitations, such as not being fully defined or ordinary. Therefore, we attempted to find new experimental conditions and related mechanisms to improve cellular estrogen responsiveness based on the standard culture medium supplemented with normal FBS and phenol red. The hypothesis of pleiotropic estrogen effects led to the discovery that T47D cells respond well to estrogen under low cell density and medium replacement. These conditions made ET less effective there. The fact that several BC cell culture supernatants reversed these findings implies that housekeeping autocrine factors regulate estrogen and ET responsiveness. Results reproduced in T47D subclone and MCF-7 cells highlight that these phenomena are general among HR+ BC cells. Our findings offer not only new insights into ET-R but also a new experimental model for future ET-R studies.

## 1. Introduction

Breast cancer (BC) is the most threatening cancer for women worldwide, ranking first in both cancer incidence (24.5%) and cancer mortality (15.5%) [1]. BCs are generally divided into three subtypes based on the expression of two or three major drivers or biomarkers: (1) hormone receptor-positive (HR+) or luminal, (2) human epidermal growth factor receptor 2-positive (HER2+) or erythroblastic oncogene B 2-positive (ERBB2+), and (3) triple-negative breast cancer (TNBC) subtypes [2].

HR+ BCs, in which HR denotes the estrogen receptor (ER) and/or progesterone receptor (PR), are the most common, accounting for approximately 80% of BCs [2,3]. Of both ERα and ERβ, ER generally refers to ERα and plays the most important role in BC development [4,5]. As most HR+ BCs, especially initially, are highly dependent on ER for growth and survival, the patients usually receive endocrine therapy (ET) or hormone therapy, which directly or indirectly inhibits ER or ER signaling pathways [4,5,6]. There are three types of ET drugs: (1) the selective ER modulator (SERM), such as tamoxifen and its derivatives; (2) the selective ER downregulator (SERD), such as fulvestrant (Fulv); and (3) the aromatase inhibitor (AI) such as letrozole, anastrozole, and exemestane [7,8]. In particular, AIs indirectly suppress ER function by inhibiting the biosynthesis of its ligands, estrogens [7]. AIs have better clinical efficacy in terms of disease-free survival, time to recurrence, or overall survival, and they have fewer side effects than tamoxifen [7,9]. Furthermore, unlike Fulv, AIs can be administered orally despite there being no superiority of AIs to Fulv in terms of efficacy and tolerability [7]. However, AIs are efficacious only in postmenopausal women or premenopausal women with suppressed ovarian function [7,10]. Therefore, since the late 2000s, AIs have been widely used as a first-line therapy for postmenopausal women with HR+ BCs [7,11].

Unlike other anticancer therapies, ET is usually given for a long period, up to ten years, and is therefore considered a preventive treatment [11,12,13]. However, ET resistance (ET-R), such as AI resistance (AI-R), occurs frequently after long-term ET [4,7]. Indeed, ET-R is much more common than expected, occurring in 30% to 50% of the patients [2,4,14]. Thus, ET-R is regarded as a major cause of cancer recurrence and metastasis, making it the most important issue in HR+ BC treatment [7,13]. The causes of ET-R include: (1) changes in the expression, activity, and sequence of ER or its coregulators such as the forkhead box A1 (FOXA1) and the proto-oncogene c-SRC (SRC); (2) constitutive or ligand/estrogen-independent ER activation; (3) hyperactivation of growth factor receptors, mitogen-activated protein kinases (MAPKs), and the phosphatidylinositol 3-kinase (PI3K)/AKT (or protein kinase B)/mammalian target of rapamycin (mTOR) pathway; and (4) dysregulation of cell cycle regulators [7,15,16]. In particular, the roles of the MAPK and AKT pathways in ET-R have been extensively studied. However, their opposite roles in ER regulation exist, showing that their pathways can induce ER activation, such as constitutive activation by ER phosphorylation (e.g., on serine 118 by MAPKs and on serine 167 by AKT), or, more fundamentally, ER loss (or cellular independence from ER), leading to ET-R [7,15]. Therefore, the mechanism of ET-R is not fully understood. Nonetheless, reversing the causative changes is used to overcome ET-R in patients [17,18].

The effect of estrogen has typically been examined under a special culture condition, i.e., phenol red (an estrogen-mimetic compound at high concentrations)-free media supplemented with dextran-coated charcoal-stripped fetal bovine serum (CS-FBS) [19,20,21]. Although CS-FBS is undoubtedly a powerful tool for maximizing estrogen responsiveness, it does have some limitations. First, the properties of CS-FBS are not clearly defined. For example, in addition to estrogens, charcoal stripping non-specifically removes a wide range of lipids (such as steroids), proteins, and vitamins from serum [21,22,23,24,25]. It does not even produce the same product consistently [24]. Second, CS-FBS may still contain a non-negligible level of estrogens, particularly estrone (E1) [24,26]. Interestingly, contrary to our expectations, normal growth media already have a fairly low concentration of estrogen, approximately 2 pg/mL of estradiol in 10% FBS, compared to physiological levels of 15–350 pg/mL of estradiol in the plasma of premenopausal women [27,28]. Third, and most importantly, CS-FBS is not the usual culture condition under which most results are obtained and thus has the potential to hinder the reproducibility of the results.

Therefore, based on the standard culture medium supplemented with normal FBS and phenol red, we attempted to discover new experimental conditions that could replace CS-FBS and the underlying mechanisms involved. Eventually, we found that certain simple methods, such as using fewer cells or replacing the medium with a new one, were effective. We presume that this is because the autocrine factors of the cells reduce their responsiveness to estrogen. The current study links estrogen-responsive and general experimental conditions, and it also provides new insights into ET-R, including AI-R.

## 2. Results

### 2.1. Although CS-FBS Makes HR+ BC Cells Much More Responsive to 17β-Estradiol (E2) Than Normal FBS, It Has Some Limitations

**Figure 1:**
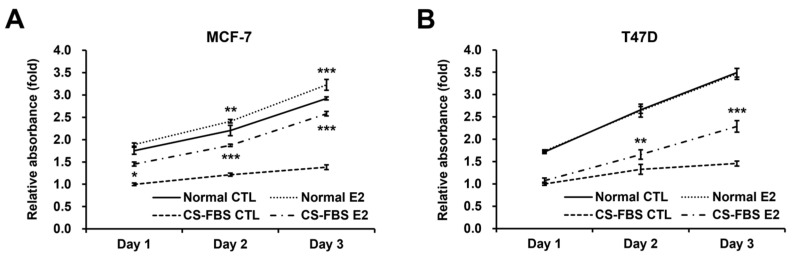
Growth curves of cell proliferation in normal fetal bovine serum (FBS) and dextran-coated charcoal-stripped FBS (CS-FBS) in the absence and presence of 17β-estradiol (E2) in two hormone receptor-positive breast cancer (HR+ BC) cell lines. MTT assay results at the relevant time after E2 treatment in MCF-7 (**A**) and T47D (**B**): cell seeding density, 0.05 × 10^6^ cells/mL; data, expressed as the mean ± standard deviation (SD) and normalized to the vehicle control (CTL) group of CS-FBS on Day 1; comparison using the two-way analysis of variance (ANOVA) test, based on each CTL at each day; * *p* < 0.05; ** *p* < 0.01; and *** *p* < 0.001.

Our two HR+ BC cell lines retained the main characteristics of HR+ mammary epithelial cells in terms of cell morphology and marker expression, despite the loss of PR in MCF-7 and weak gain of ERBB2 in T47D (Figure S1). A CS-FBS medium without phenol red made them much more responsive to E2 than a normal FBS medium with phenol red, but phenol red at a usual concentration (5 mg/mL) had only a marginal effect in normal FBS (Figure 1). Nevertheless, a sufficient supply of E2 did not completely meet the normal FBS condition in CS-FBS, as judged by cell morphology (Figures S1A and S2A). Likewise, the proliferation rate of MDA-MB-231, a representative TNBC cell line, was much lower in CS-FBS than in normal FBS (Figure S2B). These results suggest the limitations of CS-FBS. Interestingly, T47D did not respond to E2 at all under the normal culture condition (Figure 1B). Therefore, we primarily used T47D to find experimental conditions that would enhance cellular estrogen responsiveness in normal FBS.

### 2.2. Low Cell Density Increases E2 Responsiveness and Decreases Fulv Efficacy in T47D, and the Effectiveness of E2 and Fulv Decreases over Time

**Figure 2:**
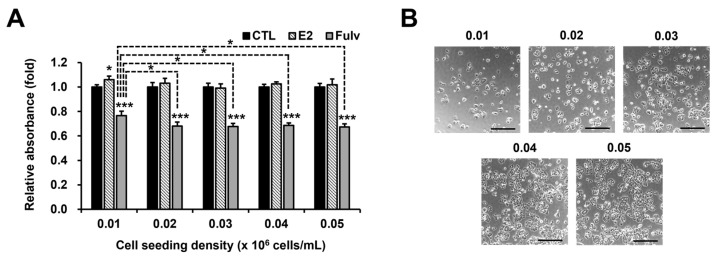
Effectiveness of E2 and fulvestrant (Fulv) on cell proliferation based on cell density in T47D. MTT assay results: data, expressed as the mean ± SD and normalized to each CTL; basic comparison (above the graph), based on each CTL; additional comparison (line), between cell densities in each reagent; statistical analysis, Student’s *t*-test; * *p* < 0.05; and *** *p* < 0.001 (**A**). Phase-contrast microscopic images (40×) of the CTLs in A just before the MTT assay: scale bar, 500 μm; the figures show how much culture space T47D occupies at each cell density (**B**).

We hypothesized that the responsiveness of HR+ mammary cells to estrogen would depend on various physiological conditions, such as cell density. Interestingly, T47D responded slightly to E2 at a low cell density of 0.01 × 10^6^ cells/mL (Figure 2). Fulv was administered in parallel with E2 to assess ER activity or cellular dependence on ER indirectly, and the antiproliferative efficacy of Fulv was remarkably reduced in reverse at the low cell density (Figure 2). Cell density affected E2 responsiveness over two passages (Figure S3A). In particular, the higher the cell density in the first passage, the greater the E2 response in the subsequent passage. When considering the actual cell density and basal cell proliferation rate in the second passage, however, this phenomenon can be explained by contact inhibition rather than the cell density itself in the first passage (Figure S3B,C). Accordingly, Figure 2 and Figure S3 indicate that only low cell density increases E2 responsiveness. In contrast, higher cell densities in the first passage did not lower Fulv efficacy in the subsequent passage (Figure S3A). Taken together, these results suggest that cell density at the time of E2 and Fulv action influences the responsiveness of T47D to E2 and Fulv, and that the mechanism of action of Fulv is not consistent with that of E2.

**Figure 3:**
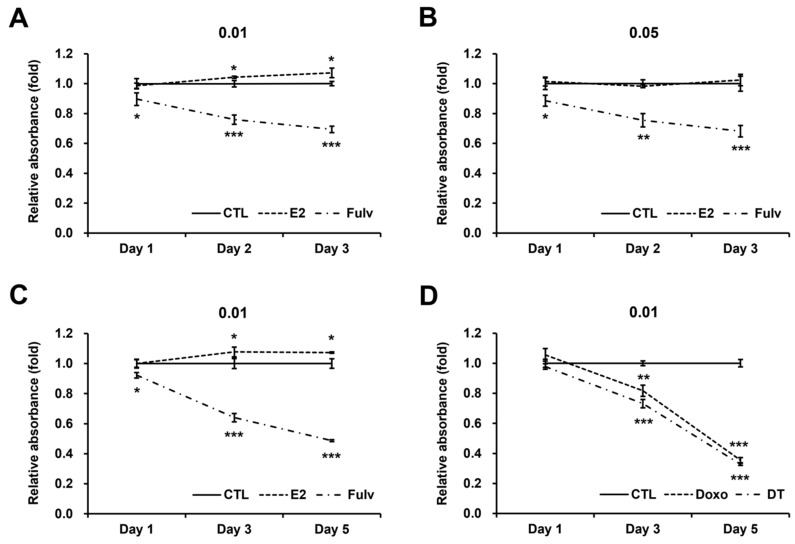
Effectiveness of E2, Fulv, and two representative chemotherapeutic agents on cell proliferation at two cell densities over time in T47D. MTT assay results for E2 and Fulv at cell seeding densities of 0.01 (**A**) and 0.05 (**B**) × 10^6^ cells/mL for up to three days. MTT assay results for E2 and Fulv at a cell seeding density of 0.01 × 10^6^ cells/mL for up to five days (**C**). MTT assay results for doxorubicin (Doxo) and docetaxel (DT) at a cell seeding density of 0.01 × 10^6^ cells/mL for up to five days (**D**). Data, expressed as the mean ± SD and normalized to each CTL at each day; comparison (above or below the graph) using Student’s *t*-test, based on each CTL at each day; Doxo, 0.1 μM; DT, 1 nM; * *p* < 0.05; ** *p* < 0.01; and *** *p* < 0.001.

E2 promoted proliferation two days after the treatment at the low cell density of 0.01, but not at the high cell density of 0.05 (Figure 3A,B). However, the growth curve slopes for days 1–2 and days 2–3 reveal that the rate of increase decreased over time (Figure 3A). This phenomenon became more noticeable over time (Figure 3C). Similar tendencies were observed with Fulv treatment (Figure 3A–C). Given that these phenomena appeared at a low enough cell density, they cannot be attributed to contact inhibition. By contrast, the potency of chemotherapeutic agents did not decrease over time (Figure 3D and Figure S4). Therefore, the temporary efficacy of E2 and Fulv, which is unusual, reveals that certain conditions necessary to sustain their efficacy become insufficient over time.

### 2.3. Medium Exchange (EXC) and Low FBS Concentration Independently Mimic Low Cell Density in Terms of E2 and Fulv Responses in T47D

**Figure 4:**
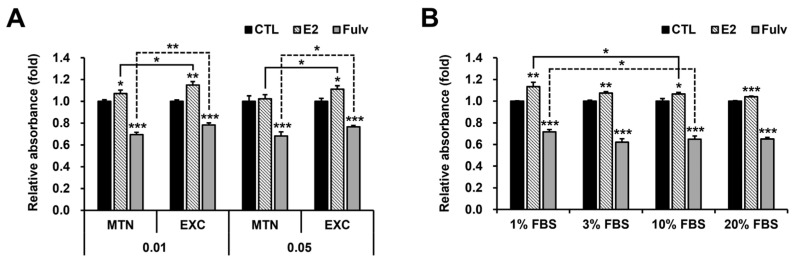
Effectiveness of E2 and Fulv on cell proliferation based on medium replacement, cell density, and FBS concentration in T47D. MTT assay results for E2 and Fulv under medium maintenance (MTN) and medium exchange (EXC) at cell seeding densities of 0.01 and 0.05 × 10^6^ cells/mL: additional comparison (line), between MTN and EXC in each reagent at each cell density (**A**). MTT assay results for E2 and Fulv based on FBS concentration: additional comparison (line), based on 10% FBS in each reagent (**B**). Data, expressed as the mean ± SD and normalized to each CTL; basic comparison (above the graph), based on each CTL; statistical analysis, Student’s *t*-test; * *p* < 0.05; ** *p* < 0.01; and *** *p* < 0.001.

To verify the time-dependent depletion or inactivation of certain components in the culture medium, the medium and reagents were exchanged for fresh ones daily. As expected, EXC increased E2 responsiveness and reduced Fulv efficacy at the low cell density of 0.01, and interestingly, similar results were observed at the high cell density of 0.05 (Figure 4A). Treatments with tamoxifen and different concentrations of Fulv reaffirmed the ET efficacy in these contexts (Figure S5). Similar results of E2 were also reproduced in phenol red-free CS-FBS and normal FBS media (Figure S6). In other words, EXC mimicked low cell density in terms of E2 and Fulv responsiveness. Under medium maintenance (MTN), each reagent was administered once at three times the concentration or added daily at the concentration for three days. Alternatively, each medium–reagent mixture was vortexed daily for three days without any additional treatment. In all three cases, there was no increase in E2 and Fulv efficacy compared to the 1× concentration and one-time treatment (Figure S7A). Similarly, even when E2 concentration was increased up to tenfold under MTN, there was no further increase in proliferation (Figure S7B). These results demonstrate that the short half-lives of E2 and Fulv and the lack of aeration do not explain their temporary efficacy, and that EXC alone is sufficient to supply the components necessary to maintain their efficacy. We conducted further experiments based on FBS concentration. Contrary to expectations, however, the tendencies were more evident at lower FBS concentrations (Figure 4B). This finding indicates that FBS does not contain the causative, labile components.

Although both intercellular adhesion and cell proliferation decreased slightly in 1% FBS compared to 10% FBS, low FBS concentrations up to 1% did not significantly affect cell properties or viability (Figure S8). Therefore, we further investigated the combined effect of 1% FBS and EXC on E2 action. Interestingly, they showed an additive or synergistic effect (Figure S9A). Although cell counting confirmed the EXC effect on E2 action in 10% FBS (Figure S9B,C), it failed to corroborate the additional effect of 1% FBS (Figure S9D,E). These results indicate that the additional effect is caused by an increase in cellular metabolism or viability, rather than cell proliferation. Cell cycle analysis also largely verified the effect of EXC in the MTT assay. Under EXC, E2 increased the S phase in both 10% and 1% FBS, but it decreased the G0/G1 phase and increased the G2/M phase only in 1% FBS (Figures S9F and S10). These cell cycle results reveal that the mechanism of action of E2 in 10% FBS differs somewhat from that in 1% FBS in the context of EXC, which is consistent with the cell count results. Therefore, careful use of the combination may be necessary. Nevertheless, the new culture condition of 1% FBS medium exchange considerably increased the responsiveness of T47D to different types of estrogens compared to the conventional condition of 10% FBS medium maintenance when using the MTT assay (Figure S11A–C). Furthermore, this new condition enabled E2 to increase cell proliferation or metabolism not only at its physiological levels in premenopausal women but also at or below those levels in postmenopausal women (Figure S11D,E) [27]. Eventually, this condition eliminated the temporary efficacy of E2 and Fulv that had appeared previously in the usual condition (Figure 3C and Figure S11F). Therefore, the combination will still be useful for maximizing estrogen responsiveness.

### 2.4. BC Cell Culture Supernatants Mimic High Cell Density and MTN in Terms of E2 and Fulv Responses in T47D, Raising the Possibility of the Involvement of Autocrine Factors in Regulating Their Responses, Although Epidermal Growth Factor (EGF) Is Not Causative

**Figure 5:**
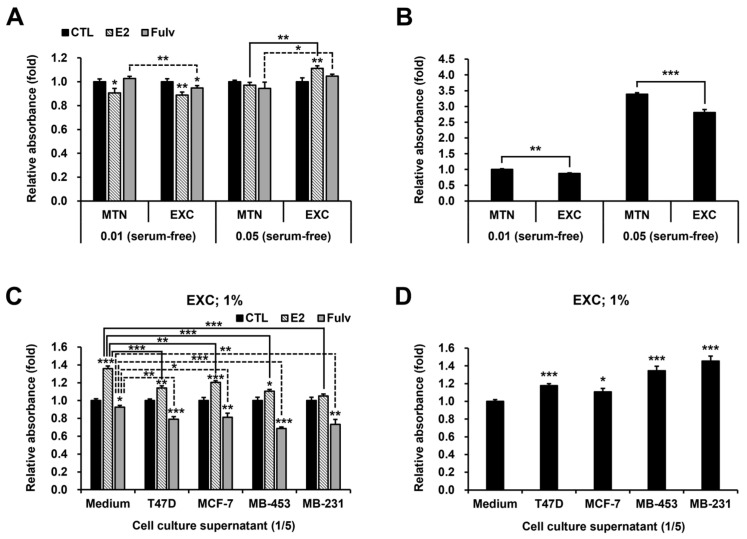
Effects of EXC and BC cell culture supernatants on E2 and Fulv actions and cell proliferation in the medium supplemented without or with FBS in T47D. MTT assay results for E2 and Fulv under MTN and EXC in serum-free RPMI 1640 at cell seeding densities of 0.01 and 0.05 × 10^6^ cells/mL: additional comparison (line), between MTN and EXC in each reagent at each cell density (**A**). Cell proliferation rates of the CTLs in (**A**): data, re-normalized to MTN at 0.01; comparison (line), between MTN and EXC at each cell density (**B**). MTT assay results for E2 and Fulv when adding 1/5 volume (compared to the preexisting medium volume) of the supernatants under EXC in the 1% FBS medium: additional comparison (line), between the medium control and each supernatant in each reagent (**C**). Cell proliferation rates of the CTLs in (**C**): data, re-normalized to the medium control (**D**). Data, expressed as the mean ± SD; data in (**A**,**C**), normalized to each CTL; basic comparison (above the graph) in (**A**,**C**,**D**), based on each control group; statistical analysis, Student’s *t*-test; * *p* < 0.05; ** *p* < 0.01; and *** *p* < 0.001.

Next, the responses were tested in the FBS-free RPMI 1640. Unlike in the FBS medium, replacing the serum-free medium did not increase E2 responsiveness or decrease Fulv efficacy at low cell density, despite the expected results at high cell density (Figure 5A). These results suggest that our previous hypothesis is incorrect. By changing our point of view, we examined the cell proliferation rate. Contrary to expectations, EXC did not promote but rather suppressed proliferation regardless of the presence of serum (Figure 5B and Figure S12A). These results indicate that certain substances secreted by the cells promote their proliferation, leading to the hypothesis that cellular autocrine factors can affect estrogen responsiveness as well as cell proliferation. As expected, increasing the medium volume slightly increased the E2 response while mimicking EXC (Figure S12B). However, there was no significant difference in E2 effectiveness between the two volumes (*p* = 0.195). This subtle difference appears to support the new hypothesis because adding the medium would only lower the concentration of autocrine factors, whereas EXC would completely eliminate the existing ones. Eventually, treating T47D with its own culture supernatant considerably decreased its E2 responsiveness and increased its Fulv responsiveness, mimicking high cell density and MTN, and interestingly, cell culture supernatants derived from three different BC cell lines all had similar effects (Figure 5C). Likewise, all the supernatants promoted proliferation, supporting the new hypothesis (Figure 5D).

T47D is an epithelial cell line in which EGF is important for cell survival, and EGF suppresses ER expression or activity in HR+ BC cells [29,30,31]. Therefore, we selected EGF as the top candidate among the putative causative factors. As expected, EGF not only promoted proliferation but also lowered E2 responsiveness (Figure S13A,B). Another control (CTL2) performed at a higher cell density (0.02) suggests that the reduced E2 response cannot be simply attributed to an increased cell number due to EGF (Figure S13B). However, EGF induced only slight changes even at a fairly high concentration compared to its physiological levels, and it did not increase Fulv efficacy (Figure S13A,B) [32]. Moreover, ELISA did not detect any additional EGF in any of the culture supernatants (Figure S13C,D). Taken together, these results suggest that autocrine factors commonly secreted by diverse BC cells not only promote cell proliferation but also modulate E2 and Fulv responsiveness, and that EGF is not among them.

### 2.5. The Results Previously Observed in the Parental T47D Cells Were Largely Reproduced in T47D Subclone and MCF-7 Cells, Albeit with Some Differences

**Figure 6:**
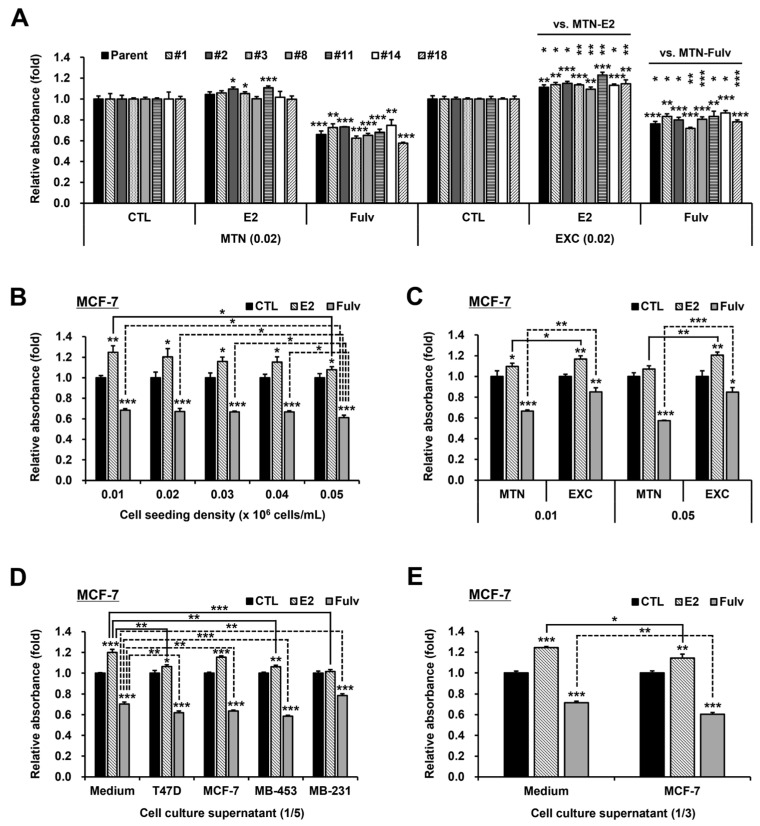
Effects of EXC, cell density, and BC cell culture supernatants on E2 and Fulv actions in single cell-derived T47D subclones or MCF-7. MTT assay results for E2 and Fulv under MTN and EXC in the T47D parental and subclone cells: cell seeding density, 0.02 × 10^6^ cells/mL; additional comparison (above the basic comparison mark), between MTN and EXC in each reagent in each subclone (**A**). MTT assay results for E2 and Fulv based on cell seeding density in MCF-7: additional comparison (line), based on 0.05 in each reagent (**B**). MTT assay results under MTN and EXC at cell seeding densities of 0.01 and 0.05 × 10^6^ cells/mL in MCF-7: additional comparison (line), between MTN and EXC in each reagent at each density (**C**). MTT assay results when adding 1/5 volume (compared to the preexisting medium volume) of the supernatants in MCF-7: additional comparison (line), between the medium control and each supernatant in each reagent (**D**). MTT assay results when adding 1/3 volume of MCF-7 culture supernatant in MCF-7: additional comparison (line), between the two groups in each reagent (**E**). Data, expressed as the mean ± SD and normalized to each CTL; basic comparison (above the graph), based on each CTL; statistical analysis, Student’s *t*-test; * *p* < 0.05; ** *p* < 0.01; and *** *p* < 0.001.

T47D cells are highly heterogeneous [33,34], so the main experiment was repeated in the single cell-derived subclones of T47D. As already known, our subclones differed from each other in terms of cell morphology and proliferation (Figure S14A,B). As a result, EXC affected the effectiveness of E2 and Fulv in all T47D subclones tested, as in the parental T47D cells, albeit to varying degrees (Figure 4A and Figure 6A). MCF-7, the most representative HR+ BC cell line, was also used. Although MCF-7 generally responded more to E2 than T47D, the effects of cell density and EXC on E2 and Fulv responses were similar for both cell lines (Figure 2A, Figure 4A and Figure 6B,C). Likewise, all the supernatants significantly decreased E2 responsiveness in MCF-7, despite the very low inhibitory potency of the MCF-7 supernatant (Figure 5C and Figure 6D,E). Except for the MDA-MB-231 supernatant, all other supernatants also increased the antiproliferative efficacy of Fulv in MCF-7 (Figure 5C and Figure 6D,E). Additionally, except for the MCF-7 supernatant, all other supernatants similarly promoted proliferation in MCF-7 (Figure 5D and Figure S15A). In MCF-7, EGF likewise decreased E2 responsiveness and promoted proliferation, but unlike in T47D, it lowered Fulv potency (Figures S13B and S15B). On the other hand, the effect of FBS concentration differed between T47D and MCF-7. In particular, the response of MCF-7 to E2 was discontinuous in relation to FBS concentration, and MCF-7 responded more to Fulv as the FBS concentration decreased (Figure 4B and Figure S16A). Moreover, MCF-7 was more dependent on serum factors for proliferation than T47D (Figures S8A,B and S16B,C). Nevertheless, an additive or synergistic effect of EXC and 1% FBS, as well as greater responses to E2 in 1% FBS than in 10% FBS, was seen in MCF-7 as in T47D (Figures S9A and S16D). Taken together, these results suggest that the aforementioned phenomena are largely common to HR+ BC cells rather than heterogeneous or cell type-specific.

### 2.6. EXC Alters E2 and Fulv Responsiveness by Modulating ER Activity Rather Than the ERα Protein Level

**Figure 7:**
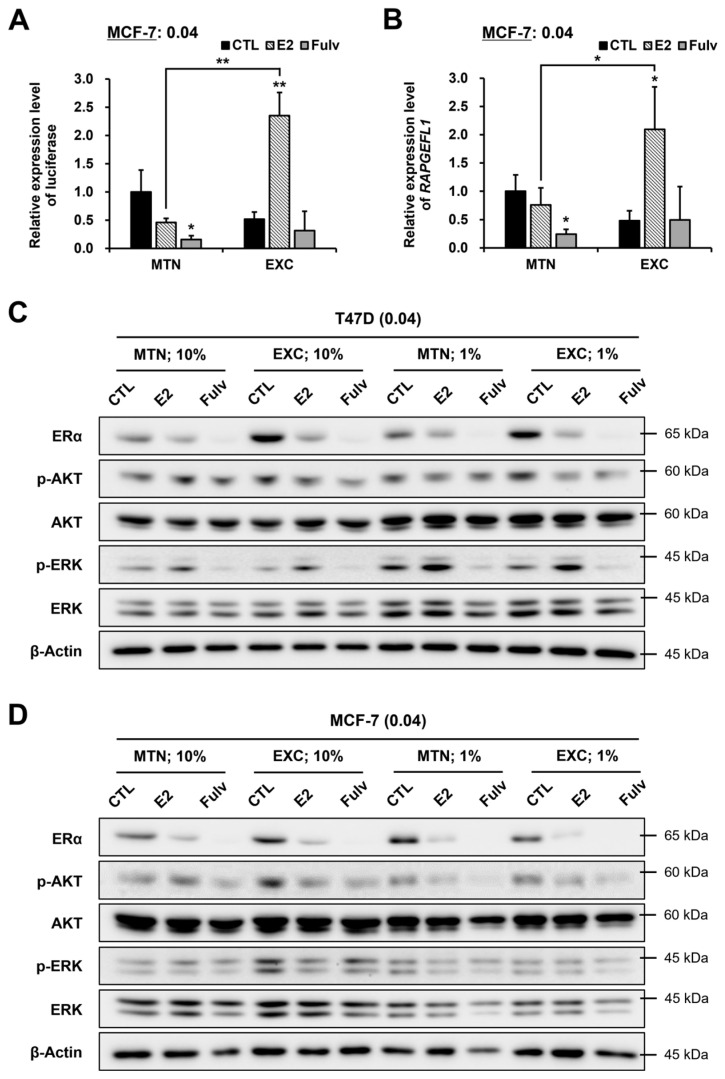
Effects of E2 and Fulv on the activity, target gene expression, or protein expression of the estrogen receptor (ER), protein kinase B (AKT), or extracellular signal-regulated kinase (ERK) under MTN and EXC in 10% or 1% FBS in MCF-7 or T47D. Results of the estrogen response element (ERE) reporter assay for the ER activity (**A**) and qRT-PCR for the expression of an ERα target gene (*RAPGEFL1*) (**B**) in MCF-7: data, expressed as the mean ± SD and normalized to CTL under MTN; basic comparison (above the graph), based on each CTL; additional comparison (line), between MTN and EXC in each reagent; statistical analysis, Student’s *t*-test; * *p* < 0.05; ** *p* < 0.01. Western blotting results in T47D (**C**) and MCF-7 (**D**). Cell seeding density, 0.04 × 10^6^ cells/mL; p-, phosphorylated.

ER activity was measured using an estrogen response element (ERE) reporter. As expected, E2 treatment considerably increased the activity and target gene expression of ER under EXC in MCF-7, in contrast to MTN (Figure 7A,B) [35]. These results reveal that EXC enables E2 to increase ERα activity, which in turn promotes cell proliferation. On the other hand, Fulv did not lower the activity and expression under EXC, unlike under MTN, similar to the results of the proliferation assay (Figure 6C, Figure 7A,B and Figure S16D). However, we were unable to confirm these findings in T47D because the ERE reporter did not function there. Western blotting showed that EXC generally increased the amount of ERα protein only at a low estrogen level (or in CTLs), except in the 1% FBS condition in MCF-7 (Figure 7C,D). These results indicate that ER activity and cell proliferation are insensitive to changes in the ER protein level, and that ER activity correlates more closely with proliferation than the ER protein level in HR+ BC cells. AKT and the extracellular signal-regulated kinase (ERK, a type of MAPK), the two most important cancer drivers, were also examined. Just like ERα, activated AKT (p-AKT) was generally increased at the protein level by EXC, whereas activated ERK (p-ERK) was not (see CTLs) (Figure 7C,D). The p-AKT levels suggest that autocrine factors also affect the AKT pathway. E2, Fulv, and EXC had no noticeable effects on cell morphology, except in the harsh 1% FBS condition in MCF-7 where EXC was favorable to the cells (Figure S17A,B). Taken together, these results suggest that certain autocrine factors influence E2 and Fulv responsiveness by regulating ER activity rather than the ER protein level in HR+ BC cells.

## 3. Discussion

We attempted to find experimental conditions and related mechanisms that would enhance cellular estrogen responsiveness based on the standard culture medium to overcome the limitations of CS-FBS. Our experimental results show that the dependence of the cells on estrogen or ER is intrinsically dependent on certain culture conditions, such as cell density and EXC. This pleiotropic effect of estrogen appears to partially explain why HR+ BCs so often survive and grow by evading ETs, such as AI treatment.

The current findings have several implications. First, it is no longer necessary to use special conditions such as CS-FBS to determine the effect of estrogen in cells. Our conditions are more natural and will contribute to better reproducibility of future findings. Second, they easily provide an “intrinsic and reversible” AI-R or ET-R model of HR+ BC cells in reverse. Third, they newly suggest that certain autocrine factors, i.e., the tumor microenvironment, influence cellular responsiveness to estrogen or ET. Fourth, experiments with different cell types indicate that our findings are generalizable. Finally, we confirmed previous findings which showed that ER activity has a stronger effect than its protein amount on both estrogen signaling and ET response. Particularly, constitutive ER activation driven primarily by post-translational modification (PTM) of ER is well known to cause ET-R [36]. Moreover, a large proportion of HR+ BCs that acquire ET-R retain ERα protein [7,37]. Likewise, we verified that the AKT pathway may be more involved in the EXC-dependent estrogen response than the ERK (MAPK) pathway. Indeed, in conjunction with ET, alpelisib and everolimus, AKT pathway inhibitors, are currently being used in patients [17,18]. However, the direct causal relationship between them needs to be studied further. Taken together, future discoveries of new intracellular or extracellular targets involved in ET-R through our new model would enable the development of novel biomarkers or combination drugs that predict or overcome ET-R in patients.

The present study has two major limitations. First, and most importantly, we could not identify the causative autocrine factors due to the overwhelming number of relevant candidates. Given that these factors may be able to function at unusually low cell densities, they are thought to be highly potent or basally secreted in very large quantities. Furthermore, as they may be commonly secreted, we believe they also act as housekeeping proteins at least in BC cells. For example, interleukins (ILs) and chemokines of the cytokine superfamily are widely involved in the development and treatment of cancers, including HR+ BC [38,39,40]. Some of these cytokines are also potent and secreted by certain cancer cells [39,41,42,43]. Thus, ILs or chemokines may be the top candidates for those factors. Although we observed no EGF secretion, other EGF family members remain candidates, as epidermal growth factor receptor (EGFR) and its other family member receptors use them [44,45]. Moreover, some of them are self-secreted by cells [44]. The multifactorial effects of the causative factors must also be addressed. It is seen as substantive evidence for this idea that only the MDA-MB-231 supernatant decreased Fulv efficacy in MCF-7. Of course, inorganic or non-peptide organic compounds produced by the cells cannot be ignored either. However, estrogen cannot be such a factor because simple treatment of intact HR+ BC cells with an AI had no antiproliferative effect.

Second, the reporter assay and qRT-PCR results cannot fully explain the consistent effect of EXC. Nonetheless, we believe that EXC consistently increases cellular estrogen responsiveness. The decreased efficacy of Fulv by EXC on ER activity and cell proliferation supports this estimation. Indeed, E2 treatment, which elevates ER activity, reduced Fulv potency. However, EXC did not increase estrogen responsiveness or ER activity in the control group. We cautiously assume that basal estrogen levels in normal FBS media are not sufficient for EXC to increase ER activity, and that Fulv has a relatively estrogen-supplementing effect by lowering overall ER activity. Transcriptome analysis is needed for a more precise mechanism. Additionally, we were unable to culture HR+ BC cells in a commercially available chemically-defined medium (11279023; Thermo Fisher Scientific), but we look forward to its successful development in the future.

## 4. Materials and Methods

### 4.1. Cell Lines and Cell Culture

All BC cells and 293FT cells were obtained from the Korean Cell Line Bank (KCLB; Seoul, Republic of Korea) and Thermo Fisher Scientific (Waltham, MA, USA), respectively. BC cells were cultured in RPMI 1640 (LM 011-01 or -02) supplemented with FBS (S 001-01; basically 10%) or CS-FBS (A3382101; Thermo Fisher Scientific) (basically 10%) and penicillin-streptomycin (LS 202-02; 0.5×) at 37 °C under a humidified 5% CO_2_ atmosphere. For 293FT, DMEM (LM 001-05) was used instead of RPMI 1640. Except for CS-FBS, all other culture reagents and cultureware (20101, 30012, and 30096) were purchased from WELGENE (Gyeongsan-si, Republic of Korea) and SPL Life Sciences (Pocheon-si, Republic of Korea), respectively. Single cell-derived subclones of T47D were established using limiting dilution cloning, as reported previously [46]. The subclones were numbered in the sequence they were established. Approximately four months after the experiment, T47D and MCF-7 were verified through DNA fingerprinting using short tandem repeat (STR) markers in the KCLB.

### 4.2. Reagents and Treatment

The reagents and manufacturers are as follows: E1 (E9750), E2 (E8875; basically used at 300 pg/mL), estriol (E3; E1253), 4-hydroxytamoxifen (4-OHT; H6278), doxorubicin (Doxo; D1515), and docetaxel (DT; 01885) from Sigma-Aldrich (St. Louis, MO, USA); Fulv (F1144; basically used at 0.1 μM) from Tokyo Chemical Industry (Tokyo, Japan); and human EGF (AF-100-15) from PeproTech (Cranbury, NJ, USA). Except for Doxo and EGF (in water), all other reagents were dissolved in dimethyl sulfoxide (DMSO; D1370) (Duchefa Biochemie; Haarlem, The Netherlands) and used as 3000× working solutions. One day after cell seeding (unless otherwise noted, at 0.01 × 10^6^ cells/mL), a 20 μL reagent-RPMI 1640 mixture was added to the used medium. Assays were performed basically three days after the treatments. For the vehicle control, only DMSO or water was used. For EXC, one day after cell seeding at the relevant FBS concentration, the existing medium was replaced with a fresh one daily for three days, and a new reagent was administered. To prepare a cell culture supernatant, cells were seeded at 0.05 × 10^6^ cells/mL in the 1% or 10% FBS medium and cultured for three days. The medium was then collected, filtered through a 0.45-μm filter (Advantec Toyo Kaisha; Tokyo, Japan), and used as is.

### 4.3. Cell Proliferation and Viability Assays

The MTT (3-(4,5-dimethylthiazol-2-yl)-2,5-diphenyltetrazolium bromide) assay was conducted to measure cell proliferation or viability [47]. MTT powder (M5655; Sigma-Aldrich) was dissolved in phosphate-buffered saline (PBS) to 5 mg/mL and further diluted 1:20 with RPMI 1640 containing 5% FBS for the working solution. For cell culture, 12-well plates were used. Cells were cooled on ice for 15 min immediately before the MTT treatment to synchronize the reaction. Cells were then exposed to 350 μL of the ice-cold MTT solution, cooled again for 5 min, and incubated in a water bath at 37 °C for 30 min. Next, the solution was removed, and the precipitate was dissolved in 350 μL of DMSO. Finally, 50–150 μL (to obtain an absorbance of ≤0.5) of the mixture was transferred to each well of a 96-well plate, and the absorbance was measured at 570 nm using a microplate reader (Molecular Devices; San Jose, CA, USA). The result value was obtained by subtracting the blank (DMSO) value from the measured value and used directly to determine the cell proliferation rate. For comparison, the relative value was calculated by dividing the result value of the reagent group by that of the control group. For the basal proliferation rates of T47D subclones, the result values on Day 4 were normalized to those on Day 1 to reduce the deviation from independent cell counting. To verify the MTT assay results, living cells were counted using a hemocytometer (Paul Marienfeld GmbH & Co. KG; Lauda-Königshofen, Germany) and trypan blue (15250-061; Thermo Fisher Scientific) according to the manufacturer’s instructions.

### 4.4. Western Blotting

The medium was removed, and cells were lysed using T-PER Tissue Protein Extraction Reagent (Thermo Fisher Scientific) supplemented with a 1× protease inhibitor cocktail (539131) (Calbiochem; San Diego, CA, USA) and 1 mM phosphatase inhibitors (sodium fluoride, sodium pyrophosphate dihydrate, and sodium orthovanadate; Sigma-Aldrich) on ice for 30 min. Cell debris was removed by centrifugation at 12,000× *g* and 4 °C for 15 min, and the protein content was quantified using BCA Protein Assay Kit (Thermo Fisher Scientific) according to the manufacturer’s instructions. For sample preparation, the lysate was diluted 3:4 with 4× Laemmli sample buffer (250 mM Tris-HCl, 4% (*w*/*v*) sodium dodecyl sulfate (SDS), 40% glycerol, 0.05% (*w*/*v*) bromophenol blue, and 4% 2-mercaptoethanol at pH 6.8) and boiled for 5 min. Next, 10 μg of protein per sample was separated on an SDS-polyacrylamide gel and transferred onto a nitrocellulose membrane using products from Bio-Rad Laboratories (Hercules, CA, USA). The membranes were then lightly blocked with 5% (*w*/*v*) skim milk dissolved in 0.05% Tween 20-containing PBS (PBS-T) and probed with primary antibodies diluted 1:1000 in PBS-T containing 3% (*w*/*v*) bovine serum albumin (BSA) at 4 °C overnight (O/N). Afterward, the membranes were probed with secondary antibodies diluted 1:5000 to 20,000 in the blocking solution at room temperature (RT) for 2 h. Membranes were thoroughly rinsed with PBS-T after each step. Lastly, the antibody-bound membranes were treated with an enhanced chemiluminescence (ECL) solution (West-Q) (GenDEPOT; Katy, TX, USA), and the luminescence was detected with the ImageQuant 800 imager (GE Healthcare; Chicago, IL, USA). Primary antibodies include: anti-ERα (sc-8002), anti-ERβ (sc-373853), anti-PR (sc-811), anti-p-AKT (sc-7985-R), anti-p-ERK (sc-7383), and anti-β-Actin (sc-47778; for the loading control) antibodies from Santa Cruz Biotechnology (Dallas, TX, USA); anti-AKT (#4691) and anti-ERK (#4695) antibodies from Cell Signaling Technology (Danvers, MA, USA); and anti-ERBB2 (06-562) and anti-p-ERBB2 (06-229) antibodies from Merk Millipore (Burlington, MA, USA). Anti-mouse and anti-rabbit secondary antibodies conjugated with horseradish peroxidase (HRP) were purchased from GenDEPOT.

### 4.5. Cell Cycle Analysis

Cells were trypsinized, harvested, fixed in 70% ethanol at 4 °C O/N, washed with PBS, and stained with 50 μg/mL propidium iodide (PI) (P1304MP; Thermo Fisher Scientific) dissolved in PBS containing 0.1 mg/mL RNase A at RT for 30 min. The cell cycle was determined using 10,000 cells with NovoCyte 3000 Flow Cytometer (ACEA Biosciences; San Diego, CA, USA). Only single cells were counted by utilizing a direct proportion of the forward scatter area (FSC-A) and forward scatter height (FSC-H).

### 4.6. Enzyme-Linked Immunosorbent Assay (ELISA)

ELISA was conducted with Human EGF ELISA Development Kit (LS-F31317) (LifeSpan BioSciences; Seattle, WA, USA) according to the manufacturer’s instructions. Briefly, a 96-well ELISA plate was coated with 0.25 μg/mL of the capture antibody at 4 °C for two days and then blocked with 1% (*w*/*v*) BSA dissolved in PBS at 4 °C O/N. For EGF standards, 1 μg/mL EGF stock solution was diluted to 30, 100, and 300 pg/mL with RPMI 1640 containing 10% FBS, as the 10% FBS medium was used for the cell culture supernatants. The standards and unknown samples were then diluted 1:3 with a diluent of PBS-T containing 0.1% (*w*/*v*) BSA. Next, the sample/standard, detection antibody (1 μg/mL in the diluent), and avidin-HRP conjugate (1:2000 in the diluent) were sequentially incubated in the plate at 4 °C O/N, at RT for 4 h, and at RT for 1 h, respectively. After each step, the solution was completely removed, and the plate was washed four times with PBS-T. Finally, after treatment with a ready-to-use ABTS (2,2′-azino-bis(3-ethylbenzothiazoline-6-sulfonic acid)) diammonium salt solution, the absorbance was measured using the microplate reader at 405 nm.

### 4.7. Quantitative Reverse Transcription PCR (qRT-PCR) to Measure the Activity and Target Gene Expression of ER

An ERE reporter consisting of three EREs upstream of the TATA box and its stable application to the genome had previously been established and was adopted in the present study [48,49]. First, a plasmid containing the ERE reporter was constructed through molecular cloning. Briefly, the ERE region from 5′-AGGTCACAGTGACCTGCGGAT-3′ to 5′-AGAGTCGACCTGCAGGCATGC-3′ and the luciferase gene were amplified by PCR using the plasmids of #11354 and #18964 (Addgene; Watertown, MA, USA), respectively. Then, the ERE promoter and luciferase gene fragments were inserted together into the region between the *Spe*I and *Bam*HI sites (the CMV promoter removed) of a lentiviral backbone plasmid of pCDH-CMV-MCS-EF1-Puro (System Biosciences; Palo Alto, CA, USA). The resulting plasmid product was verified using Sanger sequencing (Cosmogenetech; Seoul, Republic of Korea). Next, stable cell lines were generated as described previously [50]. Briefly, 293FT cells were cotransfected with the backbone product and the lentiviral packaging plasmids of pMD2.G and psPAX2 (Addgene) using Lipofectamine 2000 (Thermo Fisher Scientific) according to the manufacturer’s instructions. After two days, the culture supernatant containing lentiviral vectors was harvested and filtered through a 0.45-μm filter (25CS045AS; Advantec Toyo Kaisha). T47D and MCF-7 were then treated with the supernatant plus 3 μg/mL hexadimethrine bromide (Polybrene) (H9268; Sigma-Aldrich) at a multiplicity of infection (MOI) of approximately 0.3 to ensure that only one lentiviral vector would be integrated into each cell. Cells were then selected with 2 μg/mL puromycin (Thermo Fisher Scientific).

The expression level of luciferase or *RAPGEFL1*, induced by ER, was measured using qRT-PCR. Total RNA was isolated using TRIzol Reagent (Thermo Fisher Scientific), and cDNA was synthesized with amfiRivert cDNA Synthesis Platinum Master Mix (GenDEPOT) according to the manufacturer’s instructions. Afterward, qPCR was performed in the CFX96 system (Bio-Rad Laboratories) using a 10 μL reaction mixture containing 2.5 μL of 4× CAPITAL qPCR Green Mix (biotechrabbit; Berlin, Germany), 0.3 μM primers, and 2 μL of a 1:10 diluted cDNA sample. For PCR reactions, after an initial step of 3 min at 95 °C, two steps of 15 s at 95 °C and 30 s at 63 °C were repeated 45 times. The relative expression level was calculated based on the *ACTB* gene, an internal control, using the 2^−∆∆Ct^ method [51]. The qPCR primer sequences were 5′-GCAACTGCATAAGGCTATGAAG-3′ and 5′-CGTTTCATAGCTTCTGCCAAC-3′ for luciferase; 5′-ACAGCTATGAGGCTCTGGTG-3′ and 5′-ACGAACTCCAGCTCATGCAC-3′ for *RAPGEFL1*; and 5′-CGCCAGCTCACCATGGATG-3′ and 5′-TGCCCACCATCACGCCCT-3′ for *ACTB*.

### 4.8. Statistical Analysis

Data from a triplicate experiment were presented as the mean ± standard deviation (SD). To compare two groups, *p*-values were calculated by the Student’s *t*-test or two-way analysis of variance (ANOVA) test with Bonferroni post hoc analysis using GraphPad Prism 5 (San Diego, CA, USA). The *p*-value of less than 0.05 was defined as significant, and *p* < 0.05, *p* < 0.01, and *p* < 0.001 were expressed as *, **, and ***, respectively.

## 5. Conclusions

Low cell density and EXC rendered T47D HR+ BC cells more responsive to estrogen in the standard culture medium supplemented with normal FBS and phenol red. They made ET less effective there. Reversal of these results by several BC cell culture supernatants suggests that housekeeping autocrine factors regulate cellular responsiveness to estrogen and ET. Results reproduced in T47D subclone and MCF-7 cells indicate that these phenomena are general in HR+ BC cells. These findings not only provide new insights into ET-R but also a new experimental model for future studies to overcome ET-R in HR+ BC.

## Data Availability

All data are available within the article and in the supplementary materials archive.

## Supplementary Materials

The following supporting information can be downloaded at: https://www.mdpi.com/article/10.3390/ijms24119474/s1.
