# Supplementary material for: A New Culture Model for Enhancing Estrogen Responsiveness in HR+ Breast Cancer Cells through Medium Replacement: Presumed Involvement of Autocrine Factors in Estrogen Resistance
Source: Int J Mol Sci. 2023 May 30;24(11):9474. doi: 10.3390/ijms24119474 (PMC10253977; doi:10.3390/ijms24119474)
Supplement: Supplementary file 1 [file ijms-24-09474-s001.zip › ijms-2359400-supplementary.pdf]

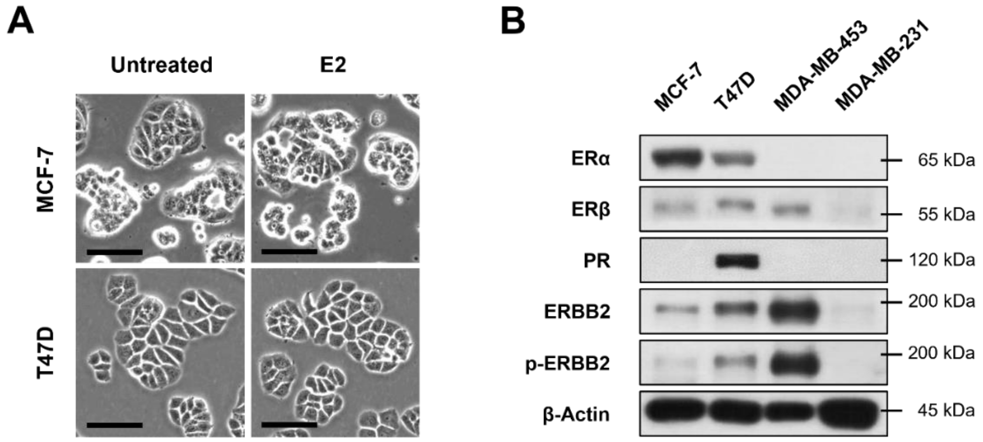

Figure S1. Cell morphology and protein levels of breast cancer (BC) subtype-specific molecular markers under normal culture conditions in MCF-7 and T47D. Phase-contrast microscopic images (100×) in the absence and presence of 17β-estradiol (E2): scale bar, 100 μm (**A**). Western blotting results: both MCF-7 and T47D, MDA-MB-453, and MDA-MB-231 are known as hormone receptor-positive (HR+) BC, human epidermal growth factor receptor 2-positive (HER2+) BC, and triple-negative BC (TNBC) cells, respectively; p-ERBB2, phosphorylated erythroblastic oncogene B 2 (**B**). Cell seeding density, 0.03 × 10^6 cells/mL.

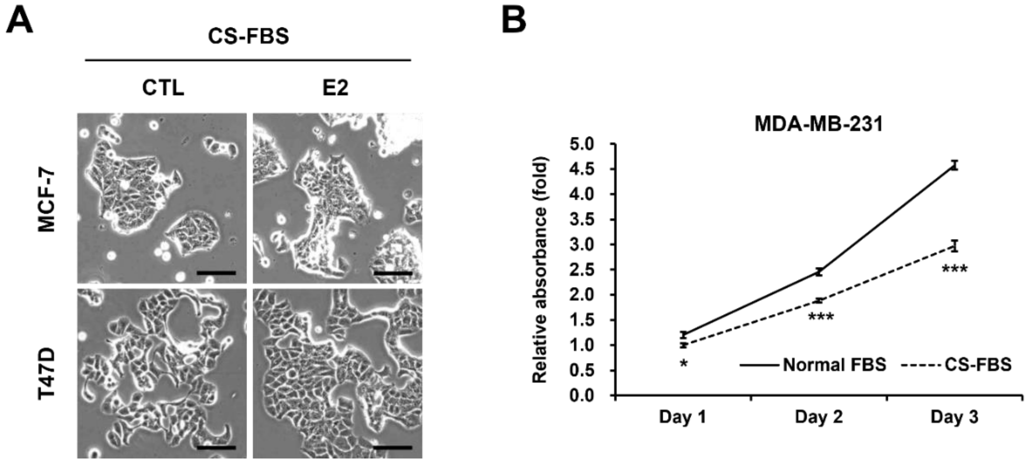

Figure S2. Cell morphology of MCF-7 and T47D in CS-FBS and growth curves of cell proliferation in normal FBS and CS-FBS in MDA-MB-231. Phase-contrast microscopic images (100×) in the absence (control; CTL) and presence of E2 at a cell seeding density of 0.05 × 10^6 cells/mL: scale bar, 100 μm (**A**). MTT assay results at the relevant time after cell seeding: cell seeding density, 0.02 × 10^6 cells/mL; data, expressed as the mean ± SD and normalized to CS-FBS on Day 1; comparison using the ANOVA test, based on normal FBS at each day; *, $p < 0.05$; and ***, $p < 0.001$ (**B**).

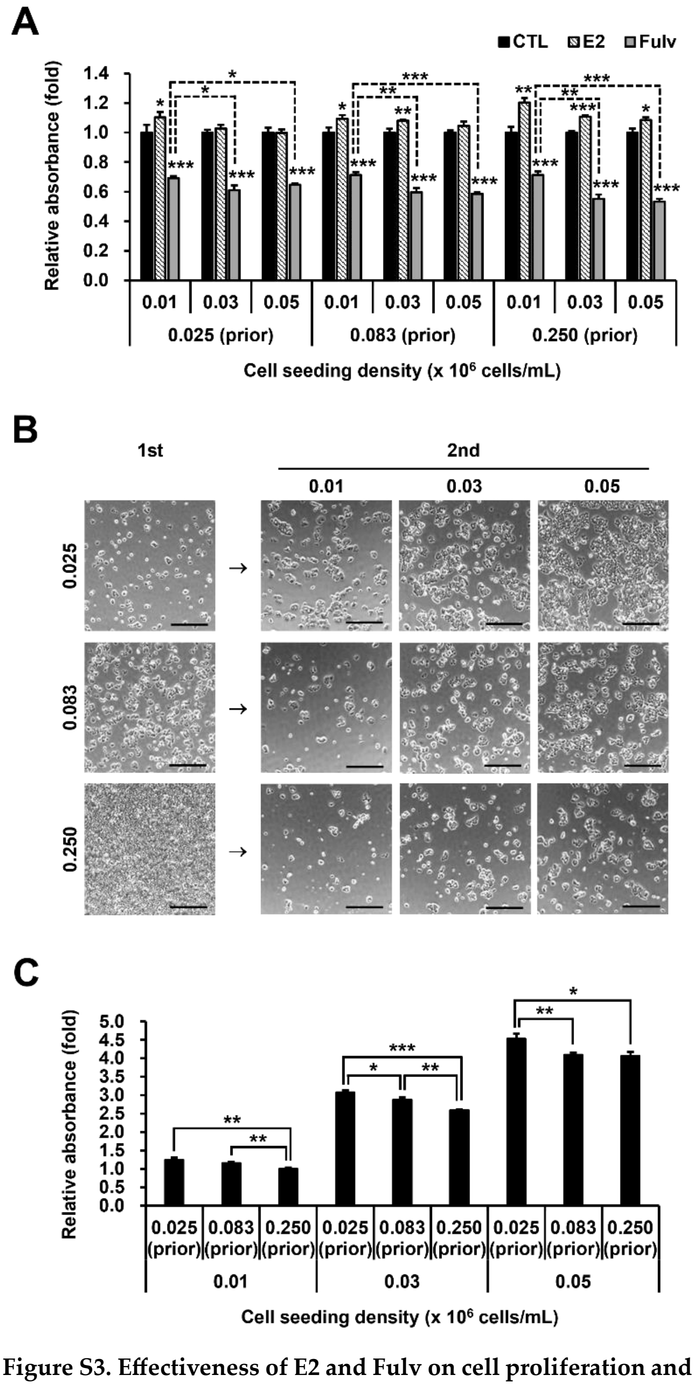

Figure S3. Effectiveness of E2 and Fulv on cell proliferation and basal proliferation rate based on cell density over two passages in T47D. MTT assay results for E2 and Fulv: after seeding cells at the relevant cell density (first, prior) and culturing them for three days, they were detached, re-seeded at the relevant cell density (second, no mark), and cultured again for one day without reagents and for three days with reagents; data, normalized to each CTL group; basic comparison (above the graph), based on each CTL; and additional comparison (line), between Fulv treatments at each density in the first passage (**A**). Phase-contrast microscopic images (40×) of the CTLs in A prior to the cell seeding or MTT assay: scale bar, 500 μm; the figures show how much culture space T47D occupies at each cell density (note that the actual cell densities of 0.025–0.01, 0.083–0.03, and 0.250–0.05 in the second passage are similar to one another) (**B**). Cell proliferation rates of the CTLs in A: data, re-normalized to the lowest result value; comparison (line), between different cell densities in the first passage at each density in the second passage (**C**). MTT data, expressed as the mean ± SD; statistical analysis, Student's *t*-test; *, $p < 0.05$; **, $p < 0.01$; and ***, $p < 0.001$.

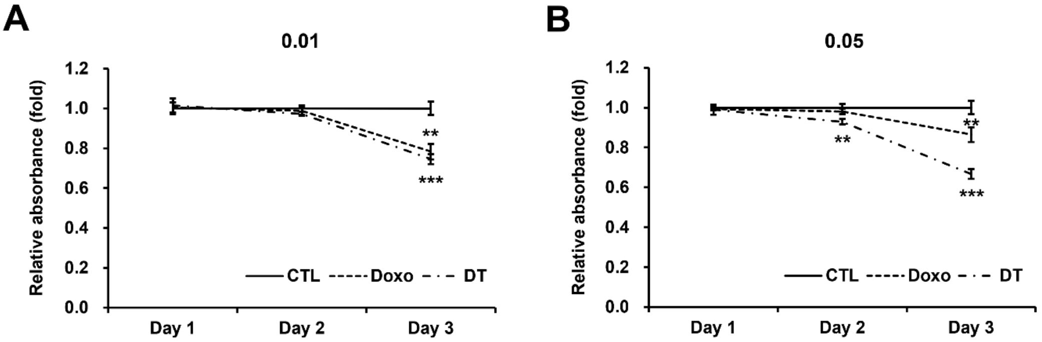

Figure S4. Effectiveness of two representative chemotherapeutic agents on cell proliferation at two cell densities over time in T47D. MTT assay results for doxorubicin (Doxo) and docetaxel (DT) at cell seeding densities of 0.01 (**A**) and 0.05 (**B**) × 10^6 cells/mL for up to three days. Data, expressed as the mean ± SD and normalized to each CTL at each day; comparison (above or below the graph) using Student's *t*-test, based on each CTL at each day; Doxo, 0.1 μM; DT, 1 nM; **, $p < 0.01$; and ***, $p < 0.001$.

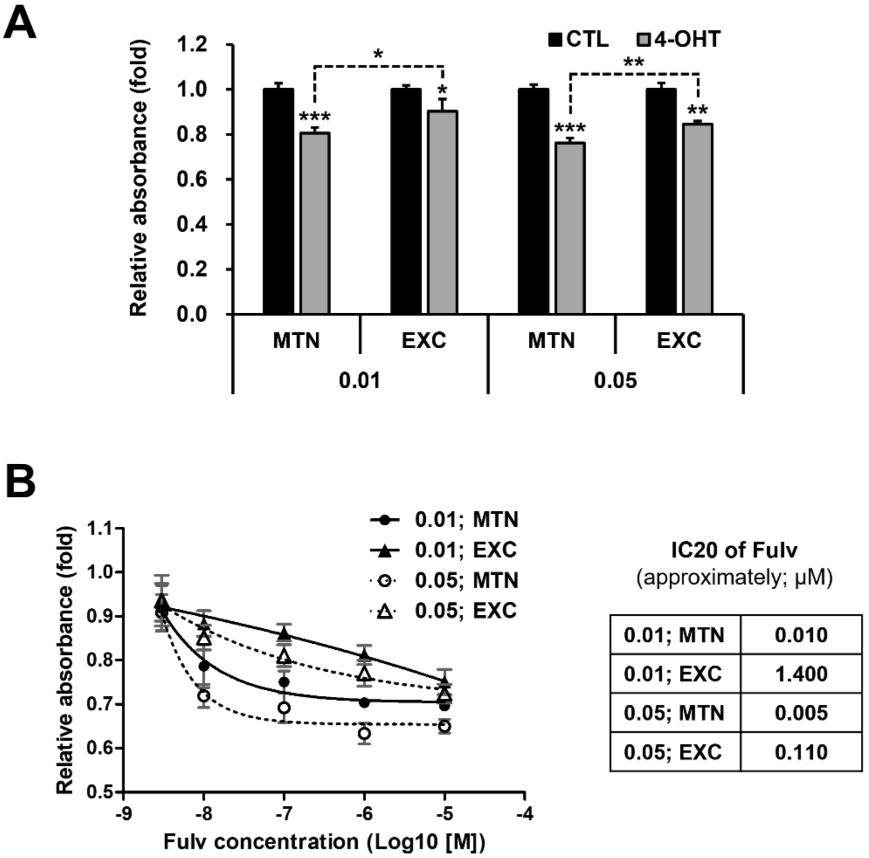

Figure S5. Effectiveness of tamoxifen on cell proliferation and dose–response curves of Fulv based on medium replacement and cell density in T47D. MTT assay results for 4-hydroxytamoxifen (4-OHT; 0.3 μM) under medium maintenance (MTN) and medium exchange (EXC) at cell densities of 0.01 and 0.05 × 10^6 cells/mL: basic comparison (above the graph), based on each CTL; additional comparison (line), between MTN and EXC at each cell density; statistical analysis, Student's *t*-test; *, $p < 0.05$; **, $p < 0.01$; and ***, $p < 0.001$ (**A**). Dose–response curves (left panel) and the 20% maximal inhibitory concentrations (IC20s; right panel) of Fulv with MTT assay results under MTN and EXC at cell densities of 0.01 and 0.05 × 10^6 cells/mL: the concentrations of 0.003 μM, 0.01 μM, 0.1 μM, 1.0 μM, and 10 μM were used; the curves were plotted using the variable slope method with GraphPad Prism 5 according to the manufacturer's instructions; and IC20s were calculated manually (**B**). Data from triplicate experiments, expressed as the mean ± SD and normalized to each control group.

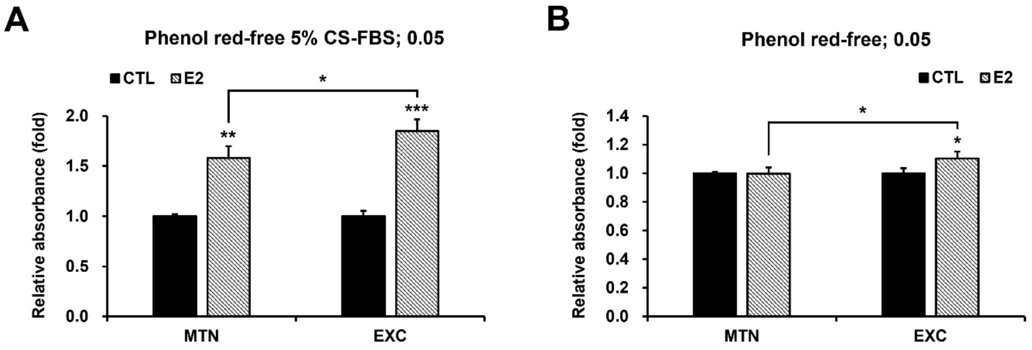

Figure S6. Effect of EXC on E2 action in terms of cell proliferation in phenol red-free CS-FBS and normal FBS media in T47D. MTT assay results for CS-FBS (5%) (**A**). MTT assay results for normal FBS (10%) (**B**). Cell seeding density, 0.05 × 10^6 cells/mL; data, expressed as the mean ± SD and normalized to each CTL; basic comparison (above the graph), based on each CTL; additional comparison (line), between MTN and EXC in E2 treatment; statistical analysis, Student's *t*-test; *, $p < 0.05$; **, $p < 0.01$; and ***, $p < 0.001$.

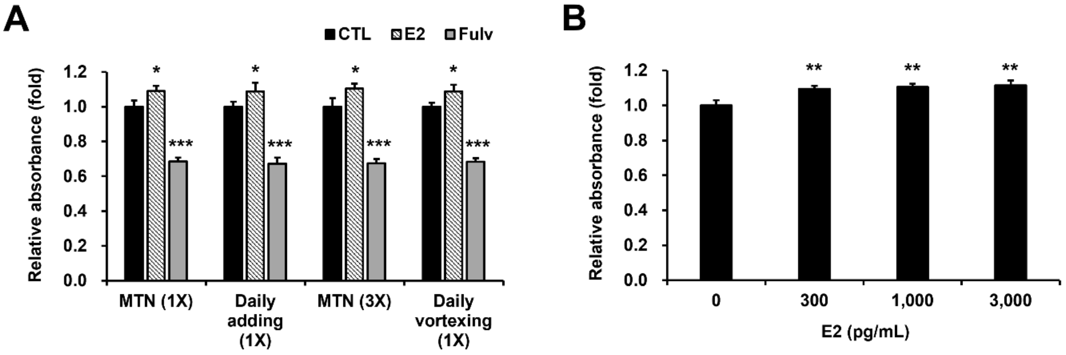

Figure S7. Effectiveness of E2 and Fulv on cell proliferation based on reagent supply and aeration in T47D. MTT assay results for E2 and Fulv based on reagent supply and aeration: MTN (1×), 300 pg/mL E2 or 0.1 μM Fulv under MTN; daily adding (1×), 300 pg/mL E2 or 0.1 μM Fulv was added daily for three days; MTN (3×), 900 pg/mL E2 or 0.3 μM Fulv under MTN; daily vortexing (1×), the reagent and medium mixture was only vortexed daily for three days; and additional comparison (no significance in all), between the experimental groups in each reagent (**A**). MTT assay results for E2 based on the concentration (**B**). Data, expressed as the mean ± SD and normalized to each control; basic comparison (above the graph), based on each control; statistical analysis, Student's *t*-test; *, $p < 0.05$; **, $p < 0.01$; and ***, $p < 0.001$.

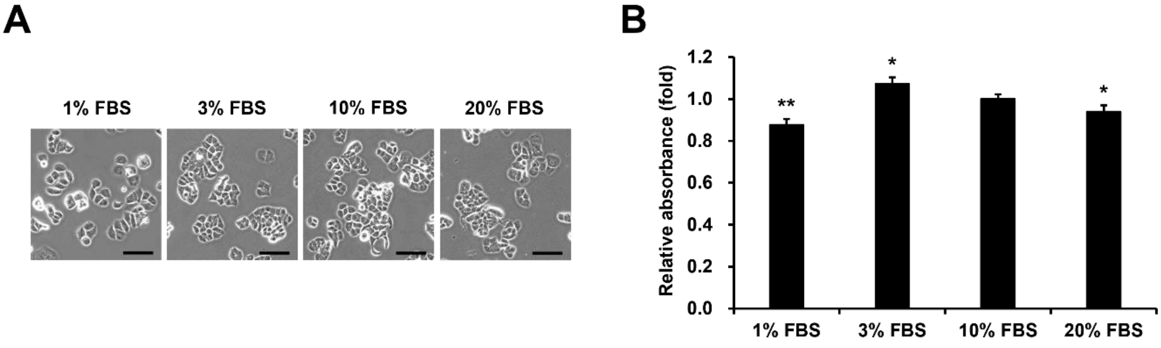

Figure S8. Cell morphology and basal proliferation rate based on FBS concentration in T47D. Phase-contrast microscopic images (100×): scale bar, 100 μm (**A**). MTT assay results: data, expressed as the mean ± SD and normalized to 10% FBS; comparison (above the graph) using Student's *t*-test, based on 10% FBS; *, $p < 0.05$; and **, $p < 0.01$ (**B**). Results were obtained four days after cell seeding.

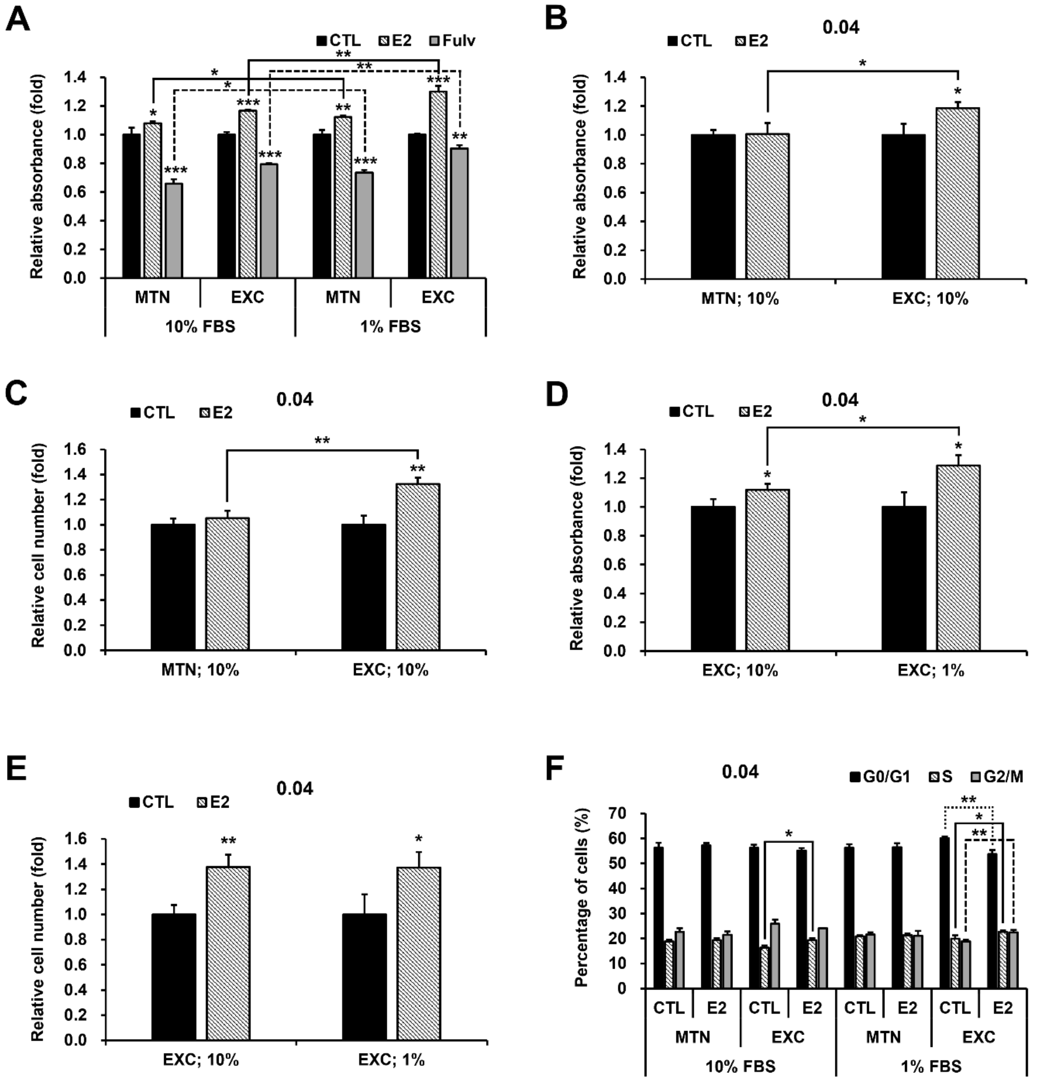

Figure S9. Effectiveness of E2 and Fulv, measured by the MTT assay, on cell proliferation under MTN and EXC in 10% and 1% FBS in T47D, and its verification by cell counting and cell cycle analysis. MTT assay results for E2 and Fulv based on medium replacement and FBS concentration: additional comparison (line), between 10% and 1% FBS in each reagent under MTN or EXC (**A**). MTT assay (**B**) and cell counting (**C**) results for E2 between MTN and EXC in 10% FBS: additional comparison (line), between MTN and EXC in E2 treatment. MTT assay (**D**) and cell counting (**E**) results for E2 between 10% and 1% FBS in EXC: additional comparison (line), between 10% and 1% FBS in E2 treatment. Cell cycle analysis results for E2 based on medium replacement and FBS concentration: basic comparison (line), between CTL and E2 in each phase under MTN or EXC; data, used as is and expressed as the mean ± SD (**F**). Cell seeding density in B–F, 0.04×10^6 cells/mL; data in A–E, expressed as the mean ± SD and normalized to each CTL; basic comparison (above the graph) in A–E, based on each CTL; statistical analysis, Student's *t*-test; *, $p < 0.05$; **, $p < 0.01$; and ***, $p < 0.001$. Both proliferation assays were performed in parallel using the same batch of samples in B and C, as well as in D and E.

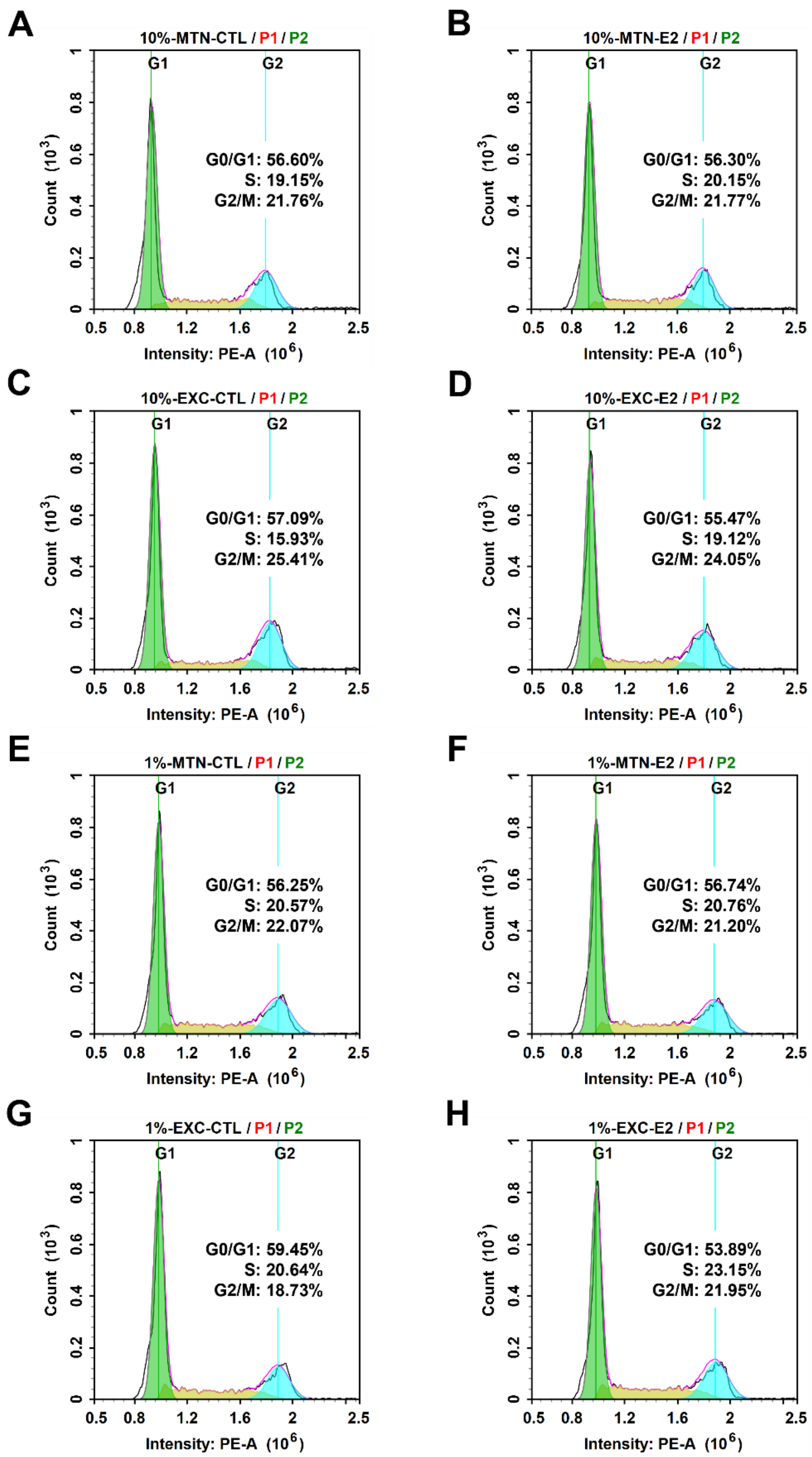

Figure S10. Raw data for cell cycle analysis shown in Figure S9F. A representative result is shown from each group: 10%, 10% FBS; 1%, 1% FBS (**A–H**). The results were rewritten in larger letters for clarity.

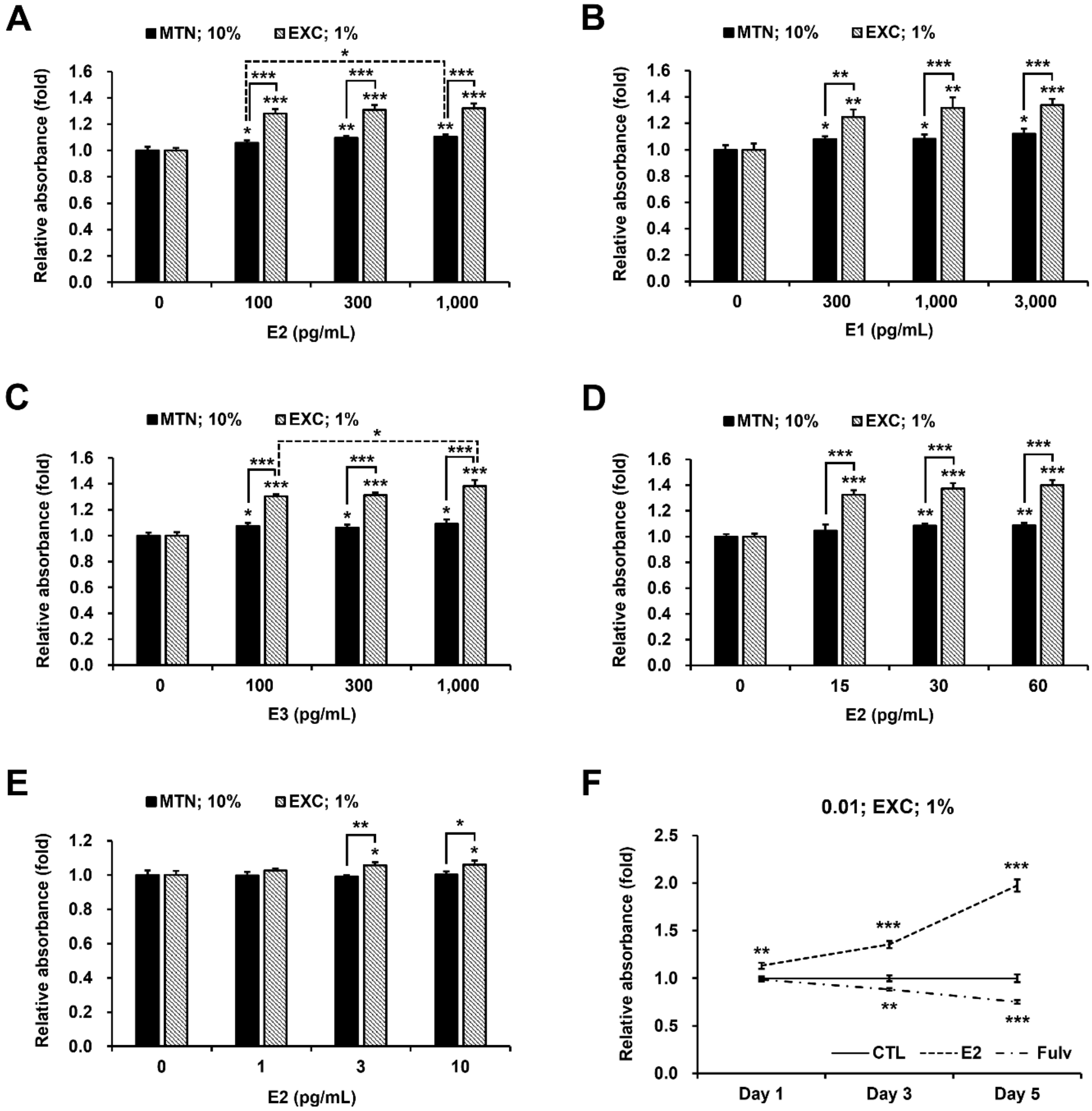

Figure S11. Effect of both EXC and 1% FBS, compared to the normal culture condition (MTN and 10% FBS), on estrogen action in terms of cell proliferation in T47D. MTT assay results for E2 (**A**), estrone (E1) (**B**), and estriol (E3) (**C**) based on estrogen concentration. MTT assay results for low levels of E2 (**D**,**E**). MTT assay results for the effectiveness of E2 and Fulv over time under EXC and 1% FBS (**F**). Data, expressed as the mean ± SD and normalized to each "0 (zero)" or each CTL; basic comparison (above or below the graph) using Student's *t*-test, based on each "0" or each CTL; additional comparison (dashed line) using Student's *t*-test, between different concentrations in each culture condition; another comparison (solid line) using the ANOVA test, between two culture conditions at each concentration; *, $p < 0.05$; **, $p < 0.01$; and ***, $p < 0.001$.

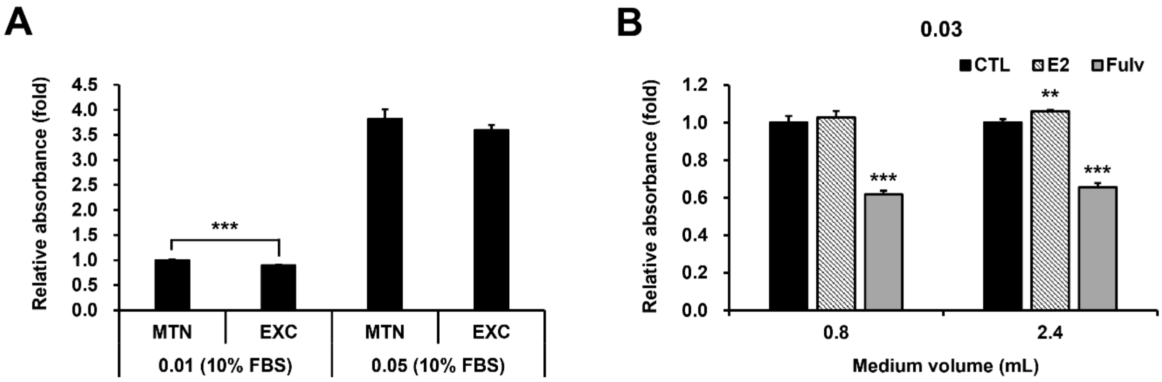

Figure S12. Effects of medium replacement and medium supply on cell proliferation, or E2 and Fulv actions in T47D. MTT assay results for the cell proliferation rates of the CTLs at cell densities of 0.01 and 0.05 × 10^6 cells/mL in the 10% FBS medium in Figure 4A: data, re-normalized to MTN in 0.01; comparison (line), between MTN and EXC at each cell density (**A**). MTT assay results for E2 and Fulv in two medium volumes: cell seeding density, 0.03 × 10^6 cells/mL; data, normalized to each CTL; basic comparison (above the graph), based on each CTL; and additional comparison (no significance), between two volumes in each reagent (**B**). Data, expressed as the mean ± SD; statistical analysis, Student's *t*-test; **, $p < 0.01$; and ***, $p < 0.001$.

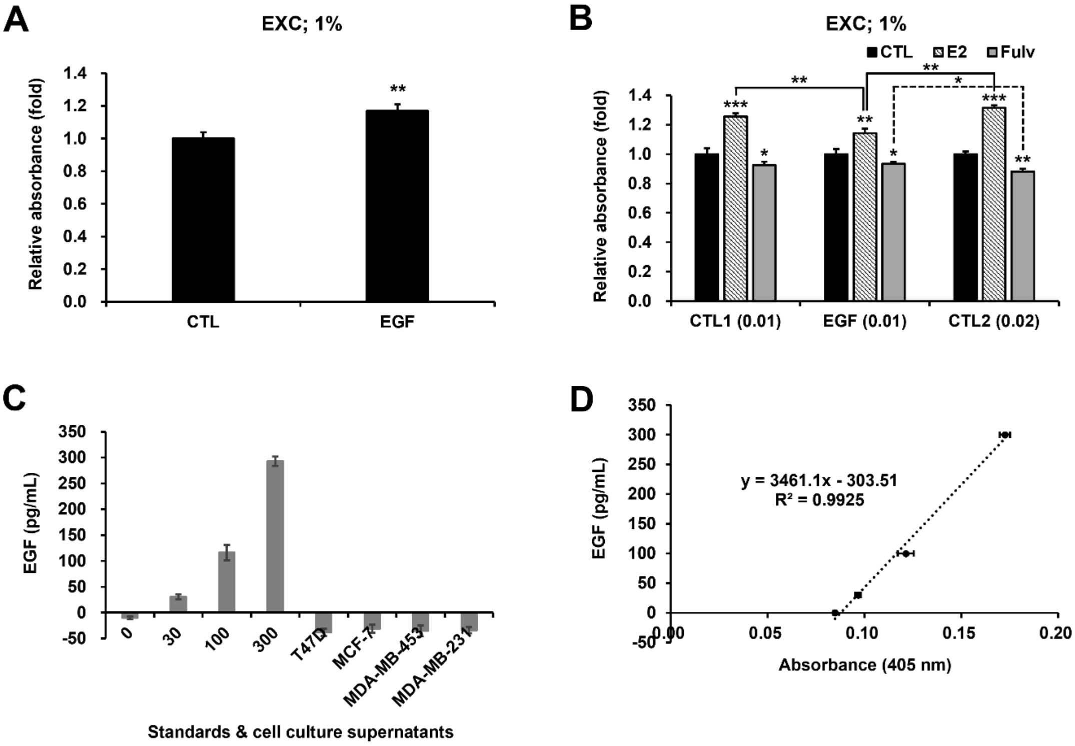

Figure S13. Effects of epidermal growth factor (EGF) on cell proliferation and the actions of E2 and Fulv in T47D, and additional or secreted EGF levels in the cell culture supernatants derived from four BC cell lines. Cell proliferation rates of CTLs in the control (CTL1) and EGF-treated groups in B: data, re-normalized to CTL in CTL1; comparison (above the graph), between the two groups (**A**). MTT assay results for E2 and Fulv in the absence or presence of 20 ng/mL EGF under EXC in the medium containing 1% FBS: cell seeding density, 0.01 or 0.02 × 10^6 cells/mL; data, normalized to each CTL; basic comparison (above the graph), based on each CTL; and additional comparison (line), based on EGF in each reagent (**B**). ELISA results of four standard and four supernatant samples: the results show "additional" EGF levels based on the fresh medium (**C**). Standard curve, deduced formula, and coefficient of determination (R2) in C: the results show the reliability of the ELISA test (**D**). Data from triplicate experiments, expressed as the mean ± SD; statistical analysis, Student's *t*-test; *, $p < 0.05$; **, $p < 0.01$; and ***, $p < 0.001$.

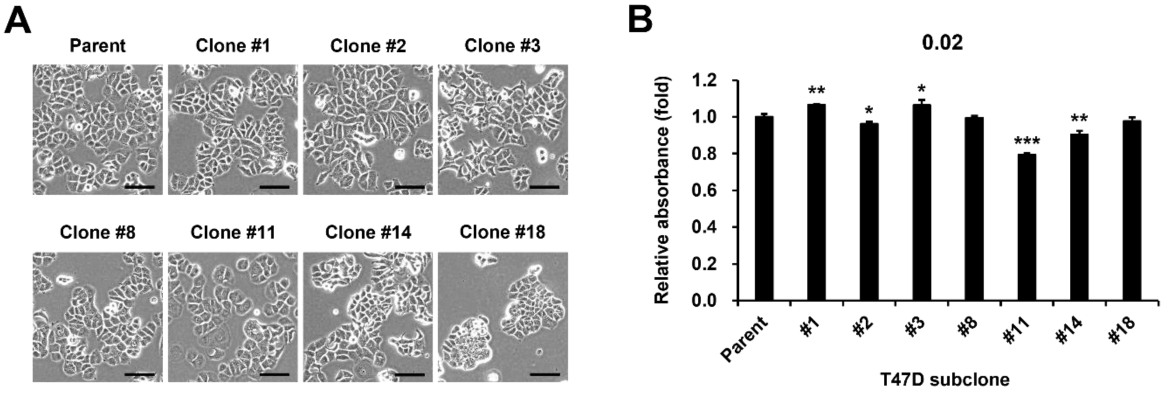

Figure S14. Cell morphology and basal proliferation rates of single cell-derived T47D subclones. Phase-contrast microscopic images (100×): scale bar, 100 µm (**A**). MTT assay results: the result values on Day 4 were normalized by dividing them by those on Day 1 to reduce the deviation from independent cell counting; data, expressed as the mean ± SD and normalized to the parental cells; comparison (above the graph) using Student's *t*-test, based on the parental cells; $p < 0.05$; **, $p < 0.01$; and ***, $p < 0.001$ (**B**). Results were obtained four days after cell seeding at 0.02×10^6 cells/mL.

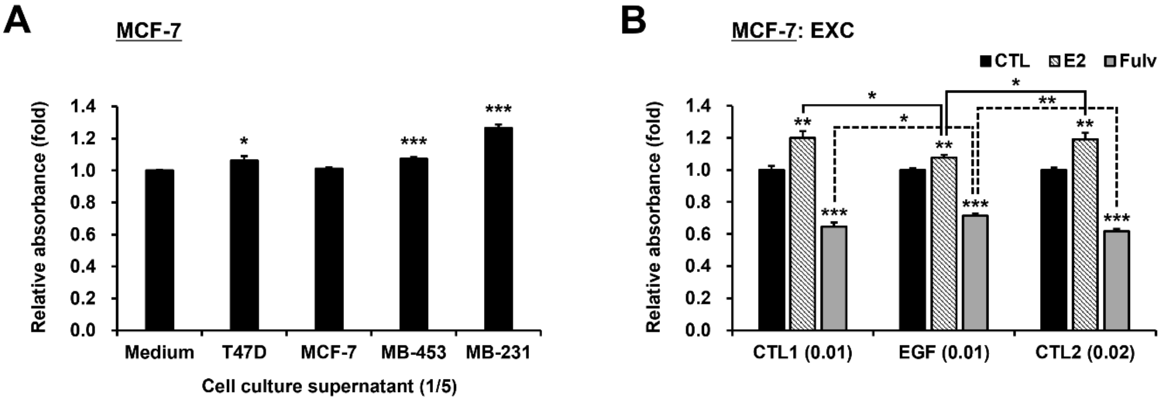

Figure S15. Effects of BC cell culture supernatants on cell proliferation and effects of EGF on E2 and Fulv actions in MCF-7. MTT assay results for the cell proliferation rates of the CTLs in Figure 6D: data, re-normalized to the medium control (**A**). MTT assay results for E2 and Fulv in the absence or presence of 20 ng/mL EGF under EXC: cell seeding density, 0.01 or 0.02×10^6 cells/mL; data, normalized to each CTL; and additional comparison (line), based on EGF in each reagent (**B**). Data, expressed as the mean ± SD; basic comparison (above the graph), based on the medium control or each CTL; statistical analysis, Student's *t*-test; *, $p < 0.05$; **, $p < 0.01$; and ***, $p < 0.001$.

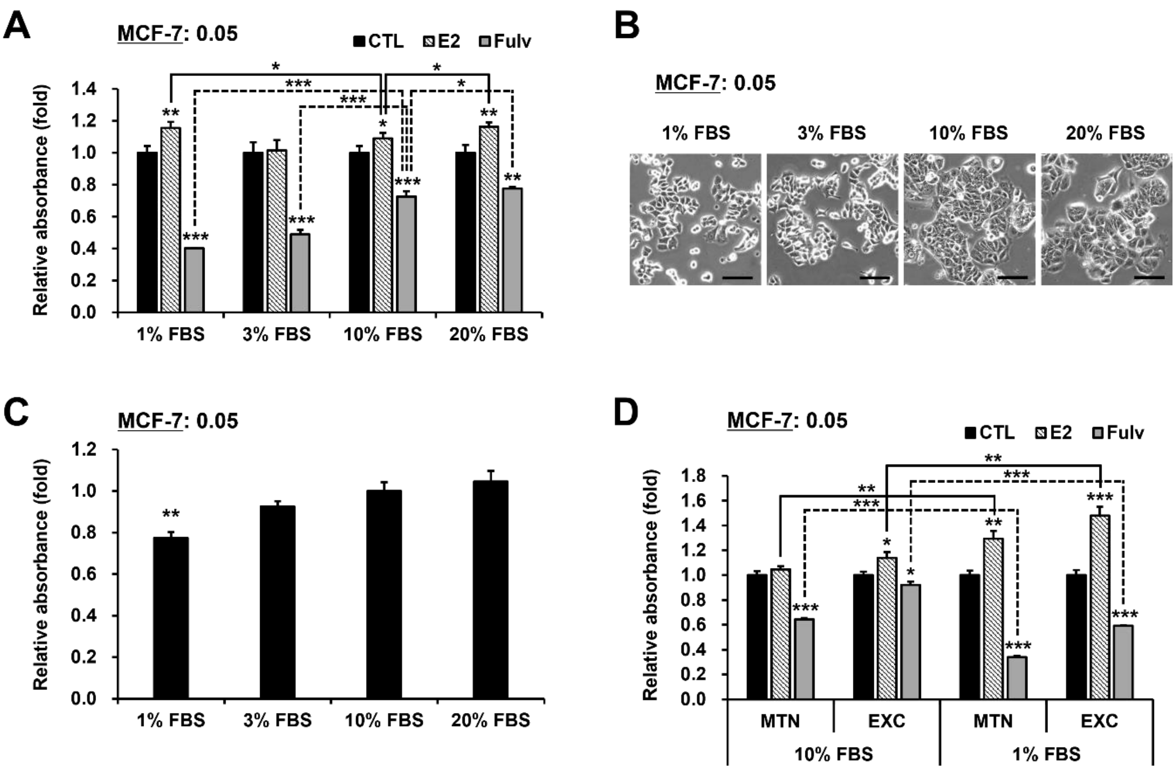

Figure S16. Effects of FBS concentration on E2 and Fulv actions, cell morphology, proliferation, and EXC effectiveness in MCF-7. MTT assay results for E2 and Fulv: additional comparison (line), based on 10% FBS in each reagent (**A**). Phase-contrast microscopic images (100×): results were obtained four days after cell seeding; scale bar, 100 μm (**B**). Cell proliferation rates of the CTLs in A: data, re-normalized to 10% FBS (**C**). MTT assay results for E2 and Fulv under MTN and EXC: additional comparison (line), between 10% and 1% FBS in each reagent under MTN or EXC (**D**). Cell seeding density, 0.05 × 10^6 cells/mL; data, expressed as the mean ± SD; data in A and D, normalized to each CTL; basic comparison (above the graph), based on each CTL or 10% FBS; statistical analysis, Student's *t*-test; *, $p < 0.05$; **, $p < 0.01$; and ***, $p < 0.001$.

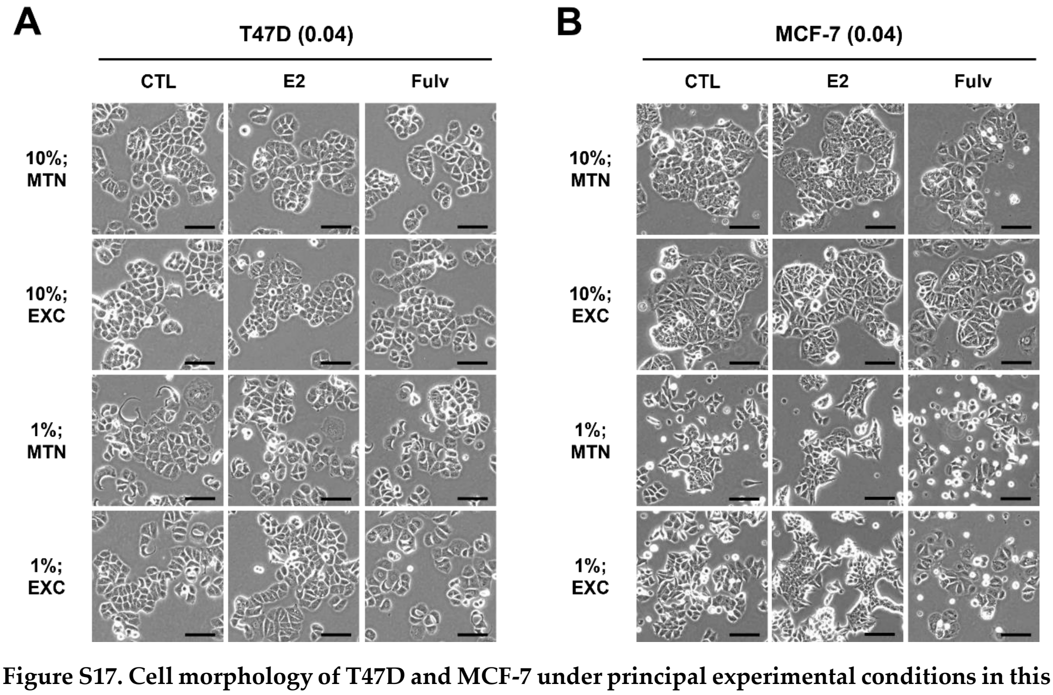

Figure S17. Cell morphology of T47D and MCF-7 under principal experimental conditions in this study. Phase-contrast microscopic images (100×) in 10% and 1% FBS in T47D (**A**) and MCF-7 (**B**): cell seeding density, 0.04 × 10^6 cells/mL; scale bar, 100 μm.
